# A Meta-Analysis of Intracortical Device Stiffness and Its Correlation with Histological Outcomes

**DOI:** 10.3390/mi9090443

**Published:** 2018-09-06

**Authors:** Allison M. Stiller, Bryan J. Black, Christopher Kung, Aashika Ashok, Stuart F. Cogan, Victor D. Varner, Joseph J. Pancrazio

**Affiliations:** Department of Bioengineering, The University of Texas at Dallas, 800W. Campbell Rd., Richardson, TX 75080, USA; bjb140530@utdallas.edu (B.J.B.); christopher.kung@utdallas.edu (C.K.); aashika.ashok@utdallas.edu (A.A.); sxc149830@utdallas.edu (S.F.C.); vdv@utdallas.edu (V.D.V.); joseph.pancrazio@utdallas.edu (J.J.P.)

**Keywords:** intracortical implant, microelectrodes, stiffness, immunohistochemistry, immune response, neural interface response, neural interface

## Abstract

Neural implants offer solutions for a variety of clinical issues. While commercially available devices can record neural signals for short time periods, they fail to do so chronically, partially due to the sustained tissue response around the device. Our objective was to assess the correlation between device stiffness, a function of both material modulus and cross-sectional area, and the severity of immune response. Meta-analysis data were derived from nine previously published studies which reported device material and geometric properties, as well as histological outcomes. Device bending stiffness was calculated by treating the device shank as a cantilevered beam. Immune response was quantified through analysis of immunohistological images from each study, specifically looking at fluorescent markers for neuronal nuclei and astrocytes, to assess neuronal dieback and gliosis. Results demonstrate that the severity of the immune response, within the first 50 µm of the device, is highly correlated with device stiffness, as opposed to device modulus or cross-sectional area independently. In general, commercially available devices are around two to three orders of magnitude higher in stiffness than devices which induced a minimal tissue response. These results have implications for future device designs aiming to decrease chronic tissue response and achieve increased long-term functionality.

## 1. Introduction

Paralysis and limb loss pose significant personal, financial, and health burdens. Each year in the U.S. alone, there are over 17,500 cases of spinal cord injury where less than 1% achieve complete recovery [1]. The nationwide prevalence of amputees is even higher at 185,000 new cases each year [2]. To address this issue, engineers and scientists are developing a range of technologies with the intent of bypassing the damaged component of the peripheral or central nervous system, to replace or restore lost motor function [3]. State-of-the-art devices are implanted intracortically, or directly into the brain, where they can record biopotentials associated with voluntary movement [4]. Neural data can then be decoded and used to drive the movement of assistive devices and prosthetic limbs, or control stimulation for functional restoration of paralyzed limbs [5,6].

While many groups have demonstrated success resolving neural signals with intracortical probes for periods of about one year [7,8], these devices tend to lose their ability to record neural signals for longer time periods [4,8,9], limiting more widespread clinical use. While there are multiple factors influencing device performance, one prominent hypothesis for device failure pertains to a chronic immune response characterized by glial encapsulation of the device, as well as local neuronal death [10,11]. Both of these compromise stable neural recordings over time. It has been suggested that a drastic mismatch in mechanical properties between the soft brain tissue and stiff neural implant may regulate the immune response [12,13,14]. Commercially available devices are fabricated using materials with a high elastic modulus, resulting in stiff devices that create concentrations of mechanical stress at the tissue interface [15], and provoke a significant, persistent immune response.

A common goal in the neuroengineering community is the development of more biocompatible implants, which elicit a decreased tissue response, with the intent of increasing their functional lifetime. These efforts are largely divided into two groups: (1) creating devices that are significantly smaller than the state-of-the-art [16,17], or (2) fabricating devices from softer materials to bridge the mechanical mismatch at the brain-device interface [18,19]. Both approaches have yielded promising results, such as decreased neuronal death and glial encapsulation, raising the possibility that a common link exists between both approaches. Our hypothesis is that these outcomes may be attributed to a single underlying parameter hereafter referred to as stiffness (*k_b_*), a function of both the material properties and geometric dimensions of the device.

Based on the mechanics of static bodies, an implantable neural probe may be treated as a simple cantilevered beam, where the beam is fixed on one end, while a downward force is placed on the other end, causing a deflection [20]. The magnitude of the deflection is inversely proportional to the stiffness of the probe, with greater deflections associated with lower stiffness. Changes in the physical dimensions and/or the mechanical properties of the probe modulate its overall stiffness. The same is true for implantable devices. Devices with lower stiffness values, or greater flexibility, can be created by modifying the cross-sectional area (CSA) and/or by using softer constituent materials.

However, ‘stiffness’ (or ‘flexibility’) is often used synonymously to describe the softness or modulus of the implantable device or device substrate, even though stiffness must consider the contributions of device dimensions. For example, while polymer-based devices may be comprised of inherently soft materials, whether or not the device is highly flexible depends on more than just their material makeup. Instead, stiffness (*k_b_*) assessments can be made based on calculations incorporating device dimensions to determine relative flexibility as compared to commercially available and other novel devices. The novelty of this study is the recognition that histological outcomes across material and geometric properties may be correlated to a single consolidated variable, *k_b_*, as opposed to relating changes in histological outcomes to a single aspect of device design.

Here, we re-evaluated a number of studies reporting details on device design and the histological outcomes following implantation in rodent brain. The analysis draws upon studies utilizing a variety of devices fabricated from a wide range of materials and dimensions, yielding a range of stiffness values. Through quantitative analysis of previously published immunohistological images, we demonstrate that the severity of the immune response is highly correlated with device stiffness. This is a function of both elastic modulus and size, in contrast to correlations considering only modulus or cross-sectional dimensions independently.

## 2. Materials and Methods

### 2.1. Stiffness Calculations

**Figure 1:**
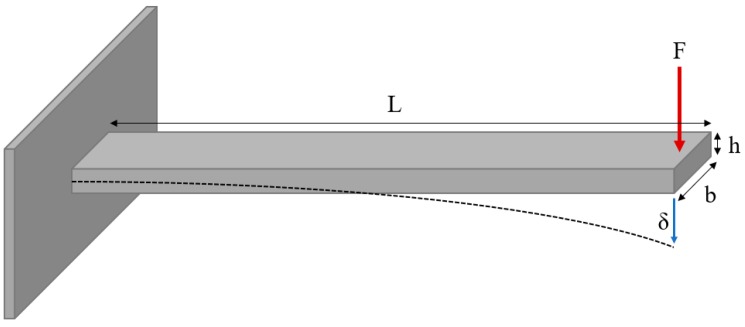
Diagram of a cantilevered beam. The beam is fixed on one end while a force on the opposite end produces a displacement, *δ*. Dimensions depicted are beam length, *L*, beam width, *b*, and beam thickness, *h*.

Table 1 lists the studies and devices used in the meta-analysis. All devices were treated as simplified cantilevered beams (Figure 1) in order to solve for bending stiffness, *k_b_*_,_ as a function of area moment of inertia, *I*, device length, *L*, and Young’s modulus, *E*, (Equation (1) [20]).

**Figure 2:**
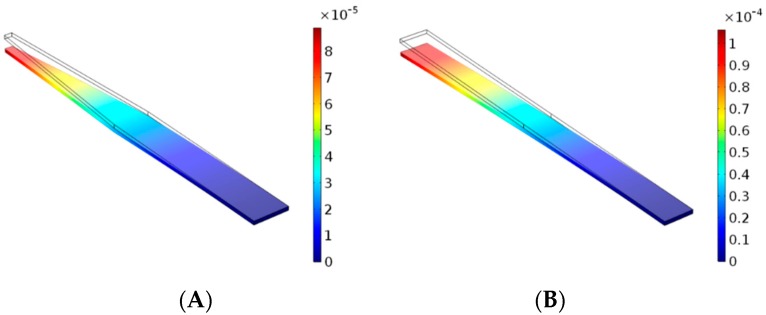
Computer simulated bend tests. Cantilever bend tests were used to determine the percent difference between device geometries with tapered and symmetrical shanks. In this case, shank (**A**) featured a width of 290 μm and tapered to 65 μm starting halfway down the shank. Shank (**B**) featured a width of 234 μm, calculated based on the weighted average of width down the length of shank (**A**). Both shanks were 30 μm thick and 3 mm long. Colored scale bars indicate deflection in meters.

Device tip geometries and shank asymmetries were neglected for the sake of simplicity. It is important to note that many single shank devices do exhibit tapered geometries meaning that cross-sectional area, and area moment of inertia, are not necessarily uniform along the length of the device. However, preliminary computational modeling suggests that using average width values does not have a significant effect on stiffness calculations. Specifically, use of a simplified symmetric model resulted in a 12% difference in maximum tip deflection in the cantilevered device bending simulation, when compared to the original tapered geometries (Figure 2). It is important to mention, however, that the tapering angle used in this simulation was relatively high when compared to those reported. Therefore, this represents a ‘worst-case scenario’ for difference in tip deflection.

Bending simulations were performed in COMSOL Multiphysics^®^ v. 5.2. (COMSOL AB, Stockholm, Sweden) and the setup mirrored the cantilever-style bend test depicted in Figure 1. Further evaluation using Linear Buckling Analysis in COMSOL revealed only an 8% difference in critical buckling force between either geometries, indicating they are mechanically similar. Therefore, representative stiffness values (Equation (1) [20]) were calculated using average values of device width along the length of the shank. For devices with polymer coatings, stiffness was assumed to be dominated by the stiffest constituent material, and calculated accordingly. Most devices did not exhibit tapering as severe as the simulation presented above; rather, this was to illustrate the worst-case scenario. As such, most of the devices exhibit less than an 8 or 12% difference in critical buckling force and maximum tip deflection, respectively. All devices were treated as having either rectangular cross sections with height, *h*, and width, *b*, or circular cross sections with diameter, *d*, affecting the way in which moment of inertia of the cross-sectional area was calculated (Equations (2) and (3) [20]). Length was taken to be the overall length of the device shank, unless the implantation depth was otherwise stated in the study.

(1) $$k_{b}=\frac{3EI}{L^{3}}$$

(2) $$I_{rectangle}=\frac{bh^{3}}{12}$$

(3) $$I_{circle}=\frac{\pi d^{4}}{64}$$

### 2.2. Image Analysis

The immune response for each study was quantified by analysis of fluorescent immunohistochemical images, from staining with several cell markers commonly associated with the immune response. For the purposes of this analysis, we focused on stains for neurons and astrocytes, specifically neuronal nuclei (NeuN) and glial fibrillary acidic protein (GFAP), a protein expressed in astrocytes.

**Figure 3:**
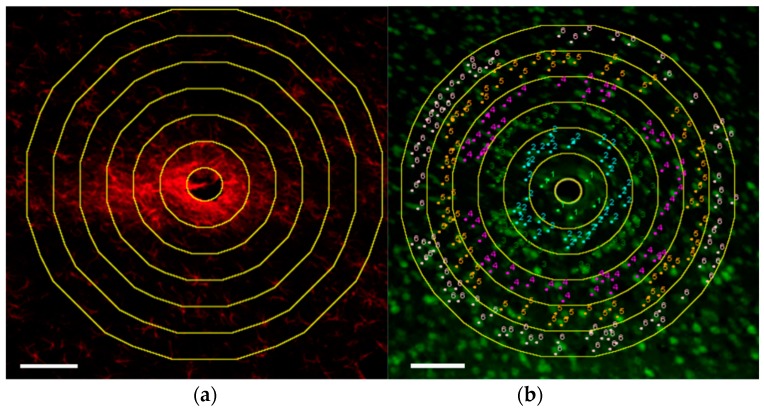
Example image analysis on a stainless steel microwire. Scale bar 100 µm. (**a**) glial fibrillary acidic protein (GFAP) intensity and (**b**) neuronal density quantification in 50 µm concentric bands. Adapted from Thelin et al., 2011 [26]. Scale bar = 100 µm.

Images were analyzed using Fiji [28], an open source image processing software based on ImageJ [29] (NIH). A custom macro was created to select the perimeter of the device within the image, and subsequently create concentric bands in 50 µm increments, while calculating the area in each band. For GFAP analysis, we computed the average intensity of GFAP immunofluorescence within each concentric band surrounding the probe (Figure 3a). For NeuN analysis, neurons were manually counted within each band using Fiji’s Cell Counter plugin to quantify neuronal density (Figure 3b). Both GFAP intensity and neuronal density were normalized by dividing each band by the value in a band at least 200–250 µm from the device perimeter. This was done to ensure normalization with respect to tissue expected to be relatively unaffected by the implant. If healthy tissue samples were provided by the study, values were normalized with respect to areas from those samples.

While several of these studies reported their own analyses of fluorescent images, we chose not to include these quantifications in this meta-analysis. This was done in order to ensure that all NeuN density and GFAP intensity values were measured and normalized consistently across all studies, for accurate comparison. However, it is important to note that studies often feature figures that best illustrate the point of the study, i.e., fluorescent images that exemplify a reduced immune response. Therefore, our results likely reflect a conservative estimate of correlations between immune markers and device parameters.

### 2.3. Statistical Analysis

To examine the possible relationships between material properties, dimensions, and device flexibility with both neuronal density and GFAP intensity, a Spearman rank correlation coefficient was calculated for each data set using functions available in MATLAB R2017a (MathWorks, Natick, MA, USA). Spearman correlation is a nonparametric test which assesses the monotonic relationship between ranked datasets. Good correlation is indicated by *ρ* values closest to 1 or 1 for positive and negative correlations respectively, with a high correlation being between 0.70 to 1.00 (or −0.70 to −1.0) [30]. A *p*-value less than 0.05 was taken as indicative of a significant correlation.

## 3. Results

**Figure 4:**
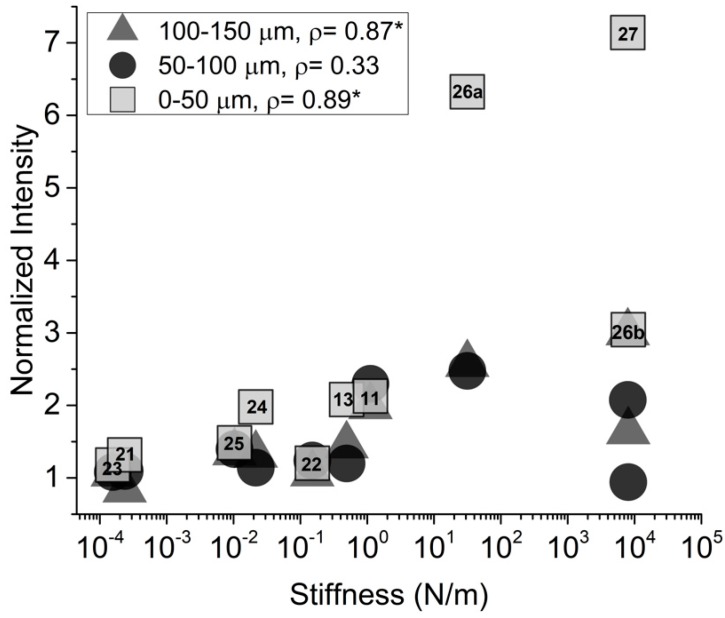
GFAP intensity analysis from GFAP-stained fluorescent images. Normalized intensity as a function of device stiffness three concentric 50 µm bands from device perimeter. * *p* < 0.05. Numbers indicate reference from Table 1. References are only included for 0–50 µm data, but all points aligned vertically are from the same reference. 26a and 26b reference the 50 µm and 200 µm stainless steel devices, respectively.

Calculated stiffness levels varied over six orders of magnitude ranging from 8 × 10^3^ to 1.6 × 10^−4^ N/m. Statistical analysis across multiple studies showed a high positive correlation (*ρ* = 0.89, *p* < 0.05) between device stiffness and normalized GFAP intensity, within a 50-µm band of the device perimeter, indicating that gliosis is more severe when using a stiffer implant (Figure 4).

Additionally, there was a high negative correlation (*ρ* = −0.92, *p* < 0.05) between device stiffness and normalized NeuN density in the same area, indicating that neuronal loss is increased when using a stiffer implant.

Device modulus and cross-sectional area did not exhibit significant correlation values within the same band, for either GFAP intensity or NeuN density, suggesting that the dependence on stiffness is a contributing factor in the severity of the immune response (Table 2). However, results also suggest that this trend is only relevant within the first 50 µm around the device. Outside of the first 50 µm band, neither GFAP intensity nor neuronal density show good, significant correlation with device stiffness, with the exception of GFAP intensity in the 100–150 µm band, therefore these data was not shown.

## 4. Discussion

**Figure 5:**
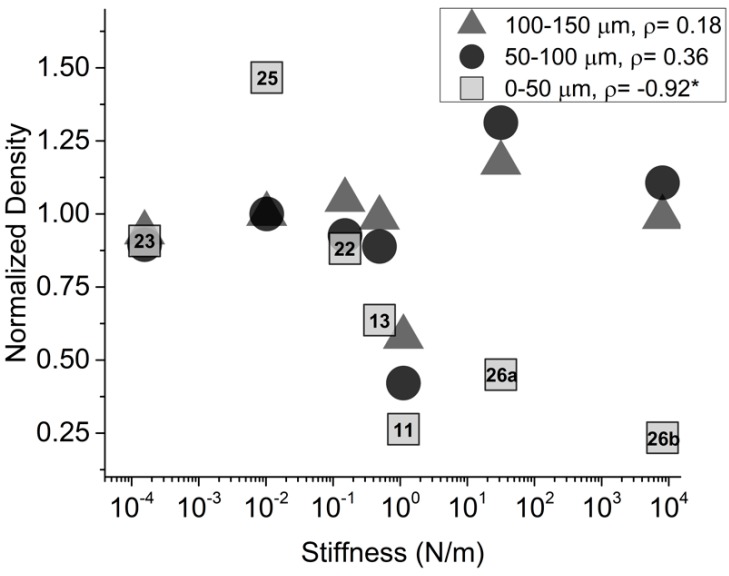
Neuronal density analysis from neuronal nuclei (NeuN)-stained fluorescent images. Normalized neuronal density as a function of device stiffness three concentric 50 µm bands from device perimeter. * *p* < 0.05. Numbers indicate reference from Table 1. References are only included for 0–50 µm data, but all points aligned vertically are from the same reference. 26a and 26b reference the 50 and 200 µm stainless steel devices, respectively.

Our meta-analysis across multiple studies indicated that the tissue response triggered during implantation may be most closely correlated with stiffness of an implanted device, as opposed to material moduli or geometric properties independently. Devices featured in this study exhibit stiffness values ranging from 10^−4^ to 10^3^ N/m. For reference, a commercially available Michigan-style silicon probe might exhibit a stiffness around 10^2^ N/m. Devices with a lower calculated bending stiffness exhibited decreased amounts of gliosis and neuronal death around the perimeter of the implant when compared with stiffer devices. These results were found to be significant within the first 50 µm of the device boundary, which is of critical importance in the context of functional neural recordings. Typically, neurons must be within 50 µm of the device electrodes in order to resolve single unit recordings at appropriate signal to noise ratios [31], and a severe immune response within this range would limit the device capabilities. Previously published studies have also reported on immune response with respect to 50 µm bands as important landmarks for histological outcomes [13,23]. Improved histological outcomes with respect to both GFAP intensity and NeuN density appeared to level off when a device reached the 10^−1^ to 10^−2^ N/m stiffness range (Figure 4 and Figure 5) indicating that this could serve as a threshold for optimal device stiffness. This stiffness could be achieved with a commercially available Michigan-style probe by reducing the thickness from 50 µm to 10 µm while maintaining an average width of 125 µm and an implantation length of 2 mm. Stiffness of tapered devices may be more accurately calculated using cantilevered setups or computational models.

Overall, high correlation between device stiffness and the severity of the immune response may be a representation of how well these devices are able to move with the brain. Cross-sectional area or elastic modulus alone do not provide a full picture: a soft object may be so large that it is stiff and cannot flex with the brain. Conversely, a small device made from a material with a high elastic modulus may face the same issue. It has been well documented that the brain experiences significant micromotion due to breathing and vascular pulsation [32]. It is likely that decreased stiffness allows these devices to move with the brain, and therefore put less strain on the surrounding tissue, perhaps leading to a less significant tissue response.

In general, these findings support approaches to changing either the material properties, or physical dimensions of devices, to reduce the severity of the tissue response. Ideally, devices featuring both soft materials and small dimensions would offer improved tissue response, but in the absence of an insertion aid, mechanical considerations must also inform the minimal stiffness required to successfully penetrate the brain. This specific limitation explains a lack of histological studies in the literature performed, using highly soft and flexible devices which would provide additional insight into the relationship between flexibility and tissue response. Additionally, very stiff devices made from high modulus materials are prone to brittle fracture, which places limits on the minimum achievable dimensions during fabrication. Furthermore, ultra-small devices have limited available surface area for electrode sites on device structures, limiting the creation of high-density probes.

The results of this meta-analysis should however encourage further exploration of materials for devices which can be fabricated in a way that limits overall stiffness (*k_b_*). This can be done through a reduction of material modulus (*E*) or a cross-sectional moment of inertia (*I*), with the goal of better matching stiffness to that of brain tissue, and subsequently improving chronic integration with surrounding tissue. Additionally, the possibility remains that the immune response may be a result of cells responding to stress concentrations due to material mismatch, as opposed to stiffness of the device itself. This hypothesis could be tested directly using an approach in which probe geometries are kept constant while varying material stiffness, or similarly, maintaining stiffness but using varied cross-sectional geometries.

## 5. Conclusions

Intracortical device stiffness may influence the severity of the chronic immune response, more than size or material properties of the device independently. Our novel results, which draw upon findings from multiple studies, indicate that device stiffness is especially important in close proximity to the device perimeter, which may profoundly affect the ability of devices to record from nearby neurons.

## Figures and Tables

**Table 1:** Devices from studies used in meta-analysis.

Author, Year	Material	Modulus	CSA (µm^2^)	Calculated Stiffness k_b_ (N/m)	Time Implanted	Stain Analyzed
Mercanzini et al., 2008 [21]	Polyimide	2.5 GPa	4200	0.00024	1 week	GFAP
Harris et al., 2011 [13]	Nanocomposite (poly(vinylacetate) and cellulose)	12 MPa	51,200	0.49	4 weeks	NeuN and GFAP
Biran et al., 2005 [11]	Silicon	179 GPa	3000	1.12	4 weeks	NeuN and GFAP
Knaack et al., 2016 [22]	Silicon	179 GPa	1875	0.15	4 weeks	NeuN and GFAP
Lee et al., 2017 [23]	OSTE soft (thiol-ene-epoxy)	6 MPa	5600	0.00016	4 weeks	NeuN and GFAP
Lewitus et al., 2014 [24]	Agarose with carbon nanotubes	Agarose-85 MPa	8220	0.02	4 weeks	GFAP
Kozai et al., 2012 [25]	Carbon fiber	234 GPa	38	0.01	2 weeks	NeuN and GFAP
Thelin et al., 2011 [26]	Stainless steel microwire (50 µm and 200 µm diameter)	200 GPa	50 µm: 1963 200 µm: 31416	50 µm: 32 200 µm: 8080	12 weeks	NeuN and GFAP
Lind et al., 2010 [27]	Bundled tungsten microwires in gelatin	Tungsten-411 GPa	70,686	7940	6 weeks	GFAP

**Table 2:** Spearman’s rho correlation results for 0–50 μm band.

			Stiffness	Modulus	CSA
Spearman’s rho	GFAP Intensity	Correlation Coefficient	0.89 *	0.62	0.42
		Significance (two-tailed)	0.001	0.06	0.23
		N	10	10	10
	Neuronal Density	Correlation Coefficient	−0.92 *	−0.09	−0.5
		Significance (two-tailed)	0.01	0.85	0.27
		N	7	7	7

*: *p* < 0.05.
